# Synthesis of Gd_2_O_3_:Eu nanoplatelets for MRI and fluorescence imaging

**DOI:** 10.1186/s11671-015-0905-4

**Published:** 2015-05-13

**Authors:** Nabil M Maalej, Ahsanulhaq Qurashi, Achraf Amir Assadi, Ramzi Maalej, Mohammed Nasiruzzaman Shaikh, Muhammad Ilyas, Mohammad A Gondal

**Affiliations:** Center of Excellence in Nanotechnology, Research Institute, King Fahd University of Petroleum and Minerals, Dhahran, 31261 Saudi Arabia; Physics Department, King Fahd University of Petroleum and Minerals, Dhahran, 31261 Saudi Arabia; Chemistry Department, King Fahd University of Petroleum and Minerals, Dhahran, 31261 Saudi Arabia; Laboratoire Géoressouces, Matériaux, Environnement et Changements Globaux, Faculté des Sciences de Sfax, Université de Sfax, Sfax, 3018 Tunisia

**Keywords:** Rare earth nanoparticles, MRI contrast, Photoluminescent nanoparticles

## Abstract

We synthesized Gd_2_O_3_ and Gd_2_O_3_ doped by europium (Eu) (2% to 10%) nanoplatelets using the polyol chemical method. The synthesized nanoplatelets were characterized by X-ray diffraction (XRD), FESEM, TEM, and EDX techniques. The optical properties of the synthesized nanoplatelets were investigated by photoluminescence spectroscopy. We also studied the magnetic resonance imaging (MRI) contrast enhancement of T1 relaxivity using 3 T MRI. The XRD for Gd_2_O_3_ revealed a cubic crystalline structure. The XRD of Gd_2_O_3_:Eu^3+^ nanoplatelets were highly consistent with Gd_2_O_3_ indicating the total incorporation of the Eu^3+^ ions in the Gd_2_O_3_ matrix. The Eu doping of Gd_2_O_3_ produced red luminescence around 612 nm corresponding to the radiative transitions from the Eu-excited state ^5^D_0_ to the ^7^F_2_. The photoluminescence was maximal at 5% Eu doping concentration. The stimulated CIE chromaticity coordinates were also calculated. Judd-Ofelt analysis was used to obtain the radiative properties of the sample from the emission spectra. The MRI contrast enhancement due to Gd_2_O_3_ was compared to DOTAREM commercial contrast agent at similar concentration of gadolinium oxide and provided similar contrast enhancement. The incorporation of Eu, however, decreased the MRI contrast due to replacement of gadolinium by Eu.

## Background

Gadolinium is a rare earth (RE) metal that has paramagnetic properties that enhance the magnetic resonance imaging (MRI) signal [1]. Gadolinium ions have seven unpaired electrons in the valence shell and hence have a high magnetic moment suitable for MRI. Gadolinium accelerates proton relaxation and hence shortens the T1 relaxation time. Gadolinium complexes such as Gd-DTPA and Gd-DOTA are some of the most commonly used clinical MRI contrast agents [2,3]. Gadolinium is a good host material for luminescence applications due to its thermal, chemical, and photochemical stability [4-6].

The gadolinium oxide doped with Eu^3+^ (Gd_2_O_3_:Eu^3+^) is paramagnetic with attractive photoluminescence (PL) properties. It is widely used in fluorescence lamps, television tubes, biological fluorescent labeling [5,7,8], MRI contrast [9-11], hyperthermia [12], immunoassays [13,14], and display applications [15-18]. Eu^3+^-doped Gd_2_O_3_ nanoparticles are red-emitting phosphors with bright luminescence and long-term photothermal stability [19]. Gd_2_O_3_:Eu^3+^ is also a very efficient X-ray and thermoluminescent phosphor [20]. Eu^3+^-doped CaF_2_-fluorophosphate glass composites has intense IR fluorescence and is a promising candidate for IR lasers and amplifiers [21].

Gadolinium oxide and RE gadolinium oxide have been synthesized by many groups using different techniques such as sol-gel [22], polyol [23], flame-spray pyrolysis [24,25], laser ablation [26], hydrothermal [17,27,28], and direct precipitation [29].

In the present work, Gd_2_O_3_ and Gd_2_O_3_:Eu^3+^ nanoplatelets were synthesized using the simple and novel polyol chemical method. Detailed structural analysis such as field emission scanning electron microscopy (FESEM), transmission electron microscopy (TEM), and energy-dispersive X-ray EDX are reported. The photoluminescent properties of Eu^3+^-activated gadolinium oxide were investigated. Judd-Ofelt analysis was used to determine the radiative properties of the synthesized nanoparticles from their PL emission spectra. The attractive multifunctional Gd_2_O_3_ and Gd_2_O_3_:Eu^3+^ nanoplatelets were investigated form MRI contrast enhancement.

## Methods

### Synthesis of Gd_2_O_3_ and Gd_2_O_3_:Eu^3+^

All reagents were of analytical grade and were used without further purification in the experiment. In this experiment, 0.5 M gadolinium acetate (Gd(OAC)_3_) was dissolved in ethanol under continuous stirring. Then 50 wt.% polyethylene glycol (mol. wt 600) was transferred in the solution under continuous stirring. After sometime, dropwise addition of 0.1 M diethylamine was carried out into the reaction solution. For the doping purpose, 2% Eu (EuCl_3_) was transferred in the solution. The resultant solution was refluxed at 100°C for 48 h. After the reaction, the flask was cooled to room temperature. The precipitates of Gd_2_O_3_ were separated from the solution by centrifuging for 30 min with a rotation speed of 3,000 rpm and then washed using deionized water. The rinsing was repeated three to five times to totally remove organic and inorganic ions adsorbed on the surface of the product. The white grayish color product was dried in an oven at 80°C for 24 h. In order to obtain highly crystalline nature of Eu-Gd_2_O_3_, the product was further calcinated in ambient atmosphere at approximately 600°C for 12 h. Similar procedure was adopted for 5% and 10% europium (Eu) doping.

### Characterization

The synthesized products were characterized using X-ray diffraction (XRD), FESEM, TEM, PL, and MRI. The crystal structure of the synthesized nanoparticles was investigated by XRD using a (XRD Shimadzu 6000; Shimadzu, Kyoto, Japan) advance X-ray diffractometer with Cu-Kα radiation source (*λ* = 1.5418 Å). The FESEM analysis was done using (FESEM JSM-6700F). TEM analysis was done on a high-resolution transmission electron microscope (HRTEM; JEOL, Tokyo, Japan). The PL spectrum was recorded using Shimadzu spectrofluorometer (Shimadzu). The excitation source was a 150-W Xenon lamp with excitation wavelength fixed at 350 nm, and the emission monochromator was scanned in the 450 to 900-nm wavelength range. The MRI contrast enhancement due to different Gd_2_O_3_ concentrations from a commercial contrast agent, Dotarem® (Guerbet LLC, Bloomington, IN, USA), was compared to the contrast due to Gd_2_O_3_ nanoparticles and Gd_2_O_3_ nanoparticles doped with Eu (2% to 10%). The different concentrations were placed in plastic 10 ml test tubes. The test tubes were placed in a plastic test tube holder and imaged in a 3 T MRI scanner (General Electric, Fairfield, CT, USA). A pulse echo T1 sequence was used with pulse repetition rates of 20, 30, 50, 100, 200, 300, 400, 500, and 1,000 ms. The images were then analyzed in order to determine the contrast enhancement due to the nanoparticles and to obtain the T1 relaxation times.

## Results and discussion

### Structural properties

**Figure 1:**
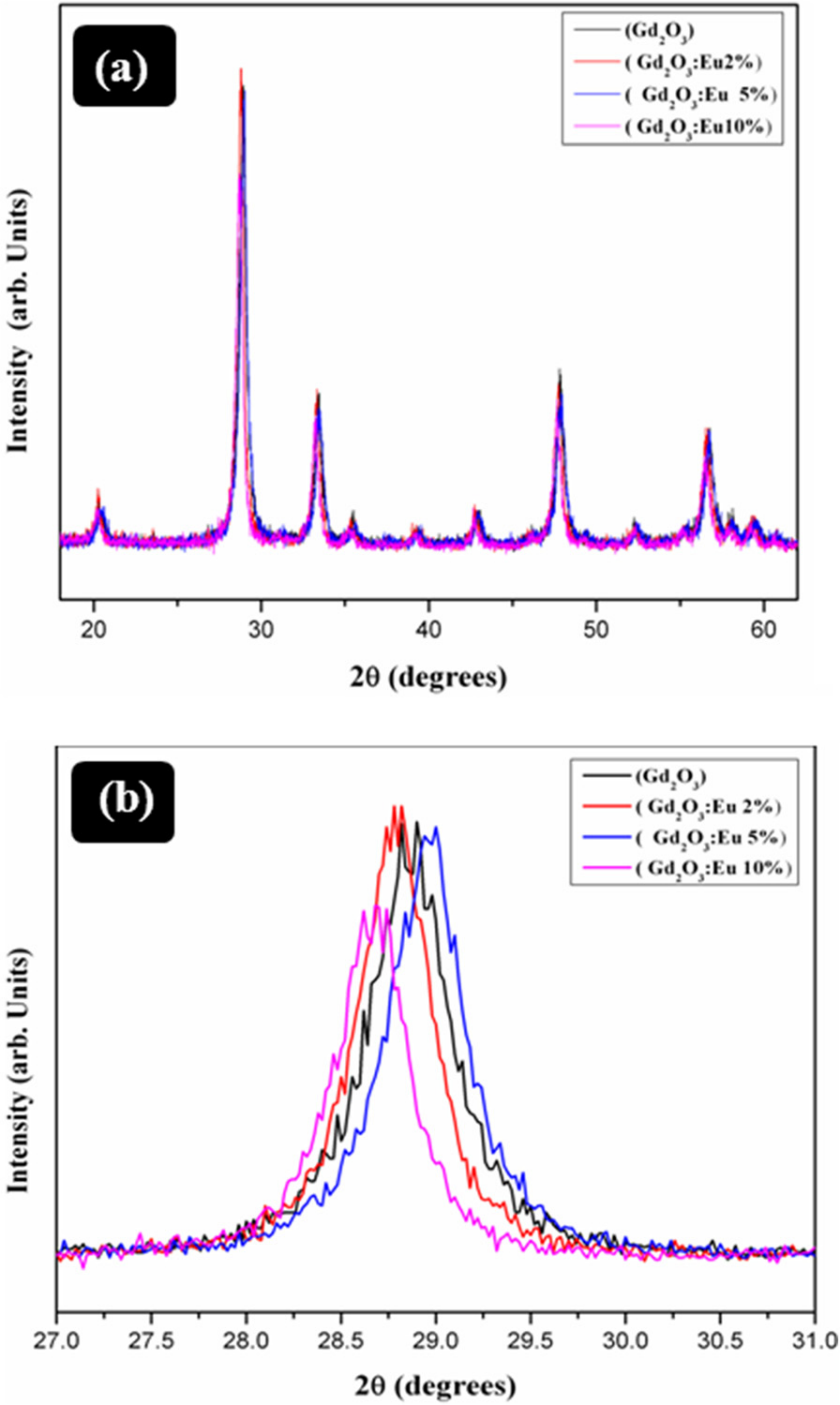
XRD pattern of Gd_2_O_3_, 2% 5%, and 10% Eu:Gd_2_O_3_ **(a)** and high-intensity (222) plane resolved for different Eu concentrations **(b)**.

XRD measurements were used to explore the phase and structure of Gd_2_O_3_ and Gd_2_O_3_:Eu^3+^ nanostructures. Figure 1a demonstrates the XRD pattern of Gd_2_O_3_ and 2%, 5%, and 10% Gd_2_O_3_:Eu^3+^, respectively. These results confirmed the cubic structure of Gd_2_O_3_ and Gd_2_O_3_:Eu^3+^ with spatial group Ia3 (JCPDS card No. 00-012-0797). No other peaks were observed in the XRD spectrum related to impurities. Due to Eu doping, a high-intensity (222) peak shift was observed as shown in Figure 1b. The presence of strong peaks indicates the highly crystalline nature of Gd_2_O_3_ nanostructures.

**Figure 2:**
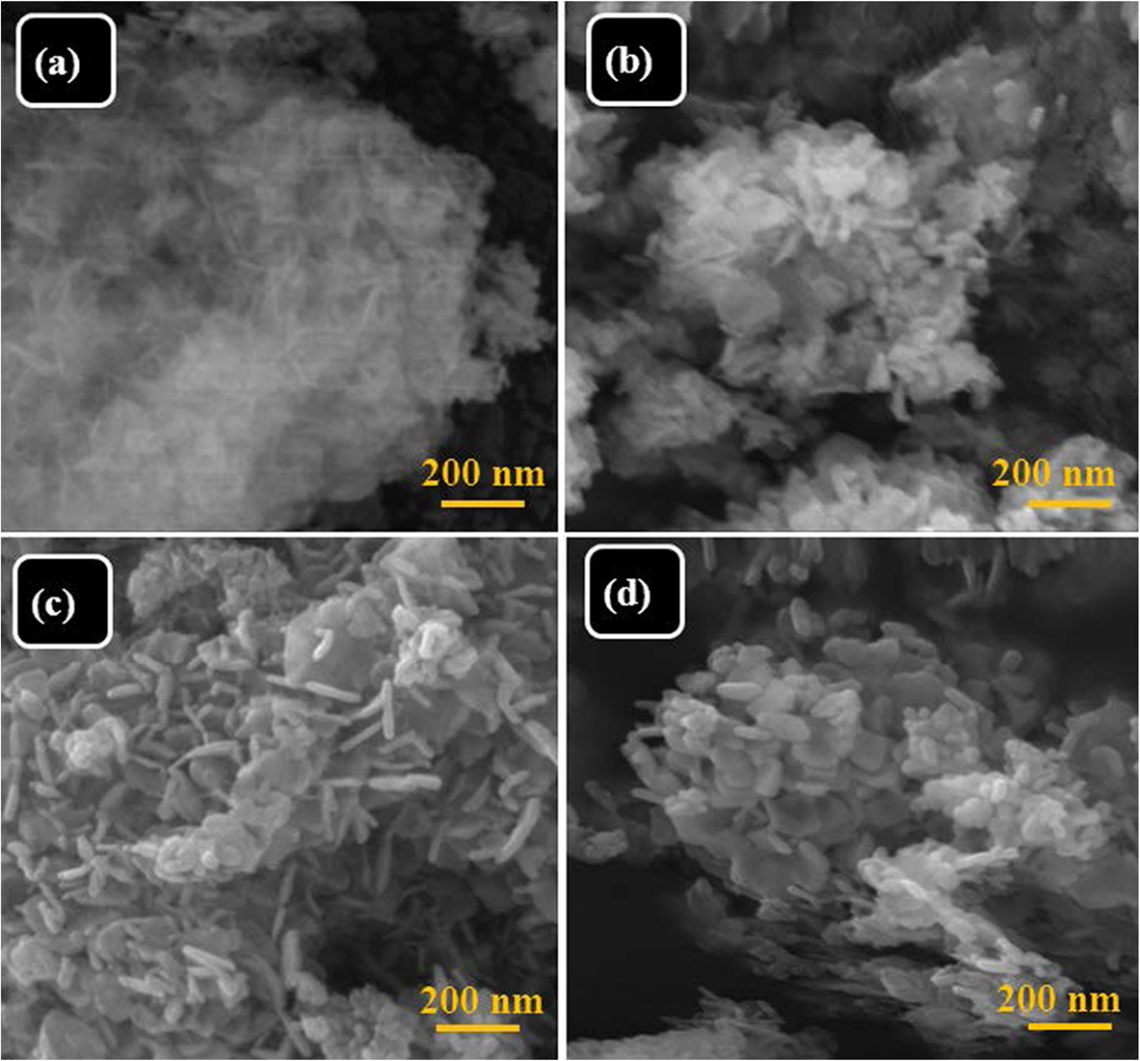
FESEM micrograph of **(a)** Gd_2_O_3_, **(b)** Gd_2_O_3_:Eu 2%, **(c)** Gd_2_O_3_:Eu 5%, and **(d)** Gd_2_O_3_:Eu 10%.

Figure 2a,b,c,d shows the FESEM images of Gd_2_O_3_ and Gd_2_O_3_:Eu^3+^ with different doping concentrations of 2%, 5%, and 10%, respectively. Figure 2a shows fine nanoflakes of Gd_2_O_3_. It is interesting to note that the thickness of the nanostructures augmented when doped with 2% Eu. Figure 2b shows FESEM micrograph of 2% Gd_2_O_3_:Eu^3+^ nanoplatelets with some nanocrystals. When the concentration of Eu^3+^ increased to 5%, highly uniform nanoplatelets were formed. The thickness of each nanoplatelet is about 15 to 25 nm (Figure 2c). Figure 2d shows FESEM micrograph of 10% Gd_2_O_3_:Eu^3+^ irregularly thick nanoplatelets. It is observed that by further increasing the concentration of Eu, the thickness and diameter of nanoplatelets increased significantly. FESEM observation showed clear change in the morphology due to doping from Gd_2_O_3_ nanoflakes to thick Gd_2_O_3_:Eu^3+^ nanoplatelets.

**Figure 3:**
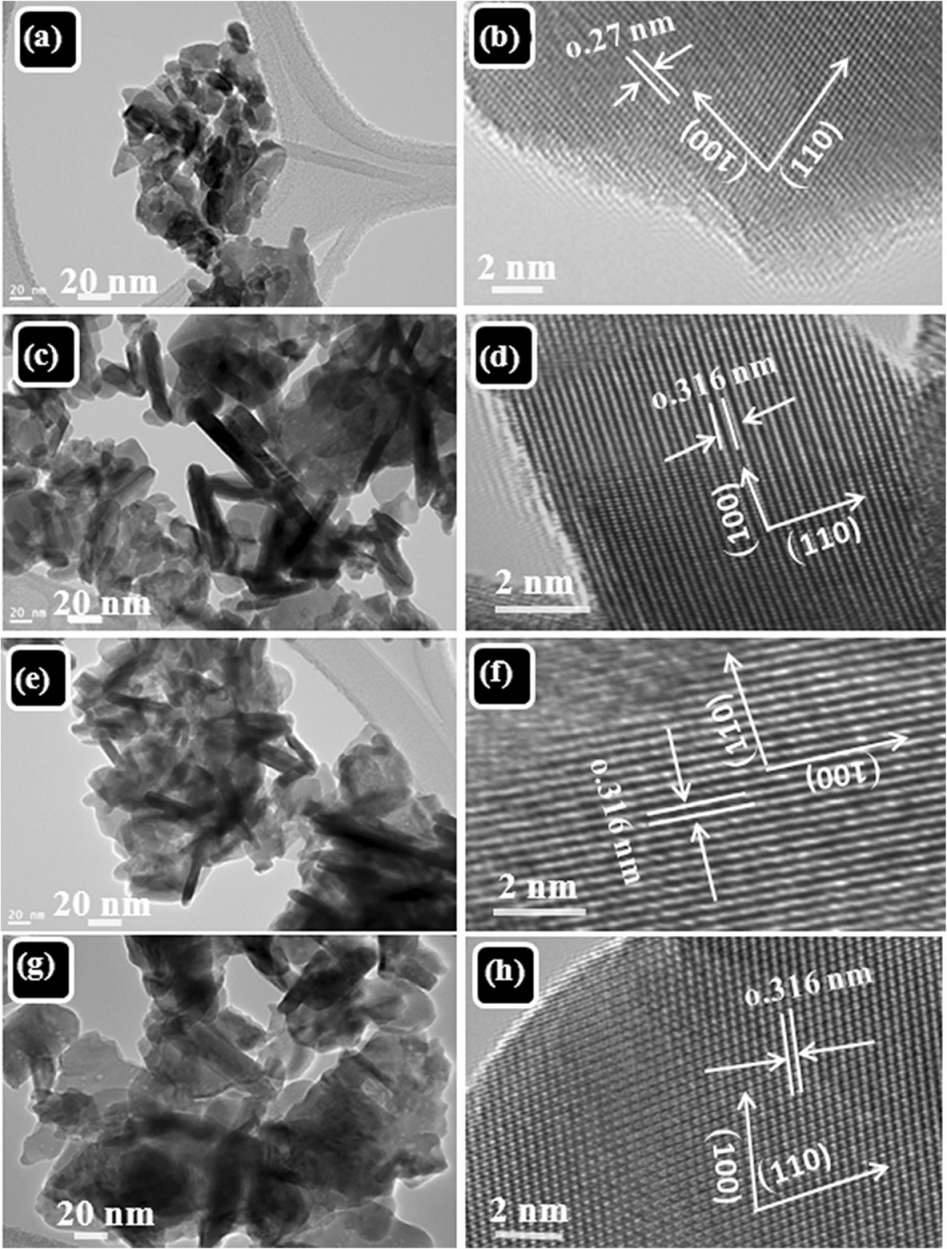
TEM micrographs of **(a,b)** Gd_2_O_3_, **(c,d)** Gd_2_O_3_:Eu 2%, **(e,f)** Gd_2_O_3_:Eu 5%, and **(g,h)** Gd_2_O_3_:Eu 10%.

**Figure 4:**
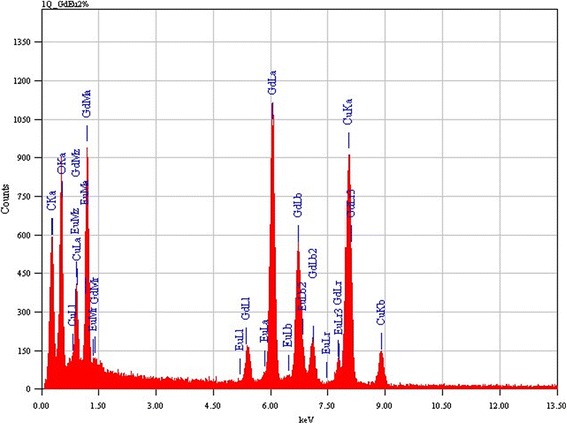
Energy dispersion spectrum (EDS) obtained from Gd_2_O_3_:Eu 2%.

Figure 3a,b,c,d,e,f,g,h shows TEM HRTEM images of Gd_2_O_3_ with different Eu concentrations (2%, 5%, and 10%). The TEM analysis is in agreement with FESEM results in which the evolution of nanoplatelets is observed. The growth of Gd_2_O_3_:Eu^3+^ nanoplatelets is seen in TEM micrographs. From the HRTEM, the interspacing between the lattice fringes was found to be 0.316 nm which corresponds to growth plane (110) indicating the growth of nanoplatelets along the axis in [001] direction. The energy-dispersive spectrum (EDS) investigation (Figure 4) also confirmed that all the detected peaks are related to Gd, O, and Eu, indicating a chemically pure Gd_2_O_3_:Eu^3+^ phase. No other peak related to impurities was found in the samples.

### Fluorescence properties

**Figure 5:**
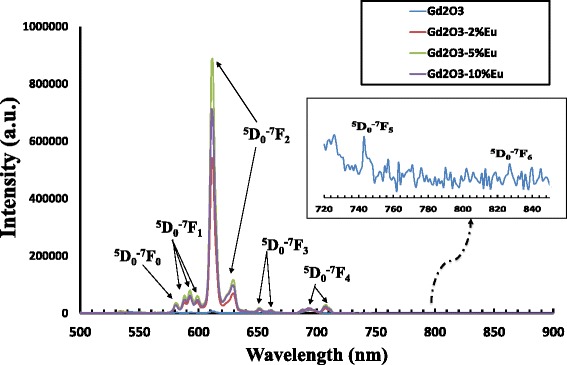
Photoluminescence spectrum of Gd_2_O_3_ and Eu (2% to 10%) doping of Gd_2_O_3_.

**Figure 6:**
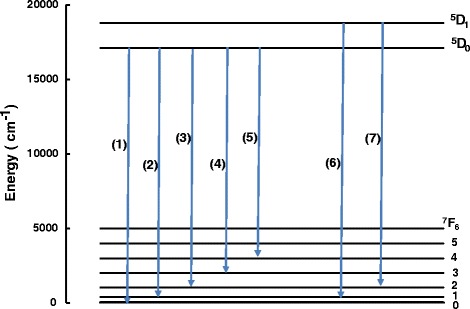
Schematic presentation of photoluminescence observed energy transitions.

Figure 5 shows the PL spectra of Eu^3+^-doped Gd_2_O_3_ nanoparticles for different dopant concentrations (2%, 5%, and 10%) recorded in the 450 to 900-nm wavelength range. The spectra have five emission lines at 580, 593, 612, 652, and 708 nm corresponding to ^5^D_0_ → ^7^F_*J*_ (*J* = 0, 1, 2, 3, 4) transitions, respectively. The two transitions corresponding to ^5^D_0_ → ^7^F*J* (*J* = 5, 6) are presented in the inset of Figure 5. We recorded a strong PL peak centered around 612 nm in addition to many smaller peaks for three different concentrations of Eu in Gd_2_O_3_. The high red luminescence signal intensity for Eu-doped samples around 612 nm corresponds to the radiative transitions from the Eu-excited state ^5^D_0_ to the ^7^F_2_ state (Figure 5). This sharp intense line indicates a complete incorporation of the dopant ions into Gd_2_O_3_ nanocrystals by replacing Gd^3+^ in a preferred C_2_ site symmetry compared to the S_6_ symmetry indicated by the ^5^D_0_ to the ^7^F_1_ transition [30]. In addition to the intense peak, numerous smaller peaks have been identified in the visible spectral range between 500 and 800 nm corresponding to the transitions from excited to the ground energy level of Eu. We also observed an increase of the emission intensities when the Eu^3+^ concentration increases to reach a maximal value at 5 mol%. Then the emission intensities decrease because of the concentration quenching. This emission behavior resembles exactly the fluorescence of Eu^3+^-doped phosphors [29,31]. Based on these measurements, we deduced an energy level scheme (Grotrian diagram) of the observed transition in PL spectra as shown in Figure 6 and Table 1.

**Table 1:** **Photoluminescence transitions observed for Gd**_**2**_ **O**_**3**_ **:Eu** ^**3+**^ **nanoplatelets**

**label**	**Wavelength (nm)**	**Transition from**	**To**
1	580	^5^D_0_ →	^7^F_0_
2	593	^5^D_0_ →	^7^F_1_
3	612	^5^D_0_ →	^7^F_2_
4	652	^5^D_0_ →	^7^F_3_
5	708	^5^D_0_ →	^7^F_4_
6	538	^5^D_1_ →	^7^F_1_
7	554	^5^D_1_ →	^7^F_2_

### CIE chromaticity coordinates

**Figure 7:**
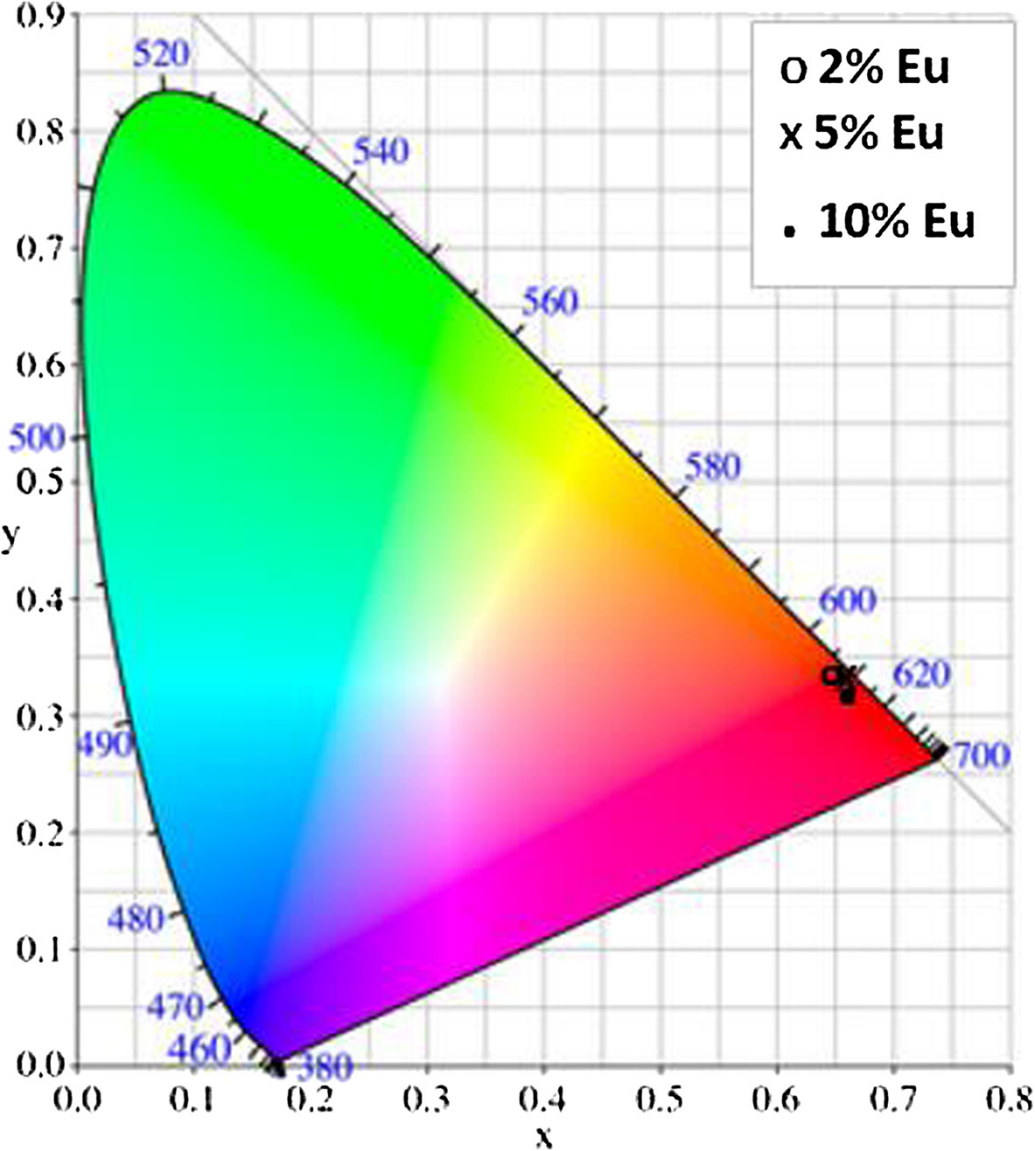
The CIE coordinate for Eu^3+^-doped Gd_2_O_3_ upon excitation at 365 nm.

The luminescent intensity of the emission spectral measurements has been characterized using the CIE1931 chromaticity diagram (Figure 7) to get information about the composition of all colors on the basis of color matching functions $\overline{x}\left(\lambda \right)$, $\overline{y}\left(\lambda \right)$, and $\overline{z}\left(\lambda \right)$ [32,33]. The (*x*, *y*) coordinates are used to represent the color and locus of all the monochromatic color coordinates. The values of the color chromaticity coordinates (*x*, *y*) were found to be (*x* = 0.6387; *y* = 0.3609) for Gd_2_O_3_:Eu^3+^ (2%), (*x* = 0. 6447; *y* = 0. 3550) for Gd_2_O_3_:Eu^3+^ (5%), and (*x* = 0. 6477; *y* = 0.3520) for Gd_2_O_3_:Eu^3+^ (10%) (Figure 7). The color coordinates are all in the pure red region of the chromaticity diagram. Indeed, the present nanoplatelets Gd_2_O_3_:Eu^3+^ give emission in the red region with appreciable intensity for fluorescence imaging.

### Judd-Ofelt and radiative analysis

The Judd-Ofelt theory [34,35] is the most widely used and known theory in the analysis of spectroscopic properties of rare earth ions in different hosts. The great appeal of this theory is the ability to forecast the oscillator strengths in absorption and to give information about the luminescence branching ratios and lifetimes by using only three parameters, Ω_*k*_ (*k* = 2,4,6) [36-39].

For the particular Eu rare earth ion-doped materials, the J-O intensity parameters are calculated with two different methods. The first method is based on the optical absorption spectra. The second method is referred to the analysis of emission spectra at room temperature. It is noteworthy to mention that in the case of Eu^3+^-doped nontransparent hosts, we are not always able to measure the absorption spectra [40,41]. Therefore, for Gd_2_O_3_:Eu^3+^ nanoplatelets, the second method allows the calculation of J-O parameters.

Table 2 shows the type of transitions for Eu^3+^ ion. The transition ^5^D_0_-^7^F_1_ is the only allowed magnetic dipole transition. The transitions from ^5^D_0_-^7^F_*J′*_ (*J*′ = 0, 3, and 5) are forbidden according to electric and magnetic selection rules. In other words, their magnetic and electrics dipoles (*A*^ed^ and *A*^md^) are zero. However, these states are not pure and are mixed with other states by crystal-field interaction, which allow these transitions to be observed as is shown in Figure 5. The transitions ^5^D_0_-^7^F_*J*′_ (*J*′ = 2, 4, and 6) are allowed electric dipole transitions and depend solely on Ω_*k*_ (*k* = 2, 4, 6).

**Table 2:** **Wavenumbers, transition rates, and branching ratio for** ^**5**^ ***D***_**0**_ **→** ^**7**^ ***F***_***J*'**_ **(** ***J*** **' = 0 − 6) of Eu** ^**3+**^ **ions in Gd**_**2**_ **0**_**3**_

**Transition**	**Type**	**Wavenumber (cm** ^**−1**^ **)**	**Transition rate (s** ^**−1**^ **)**	**Branching ratio** ***β*** **(%)**
^5^D_0_ → ^7^F_0_	Forbidden	17,241	19.1132	1.9109
^5^D_0_ → ^7^F_1_	Magnetic dipole	16,863.4	66.5612	6.6548
^5^D_0_ → ^7^F_2_	Electric dipole	16,339	876.9950	87.6820
^5^D_0_ → ^7^F_3_	Forbidden	15,337	10.0094	1.0007
^5^D_0_ → ^7^F_4_	Electric dipole	14,124	27.0115	2.7006
^5^D_0_ → ^7^F_5_	Forbidden	13,422	0.4321	0.0432
^5^D_0_ → ^7^F_6_	Electric dipole	12,422	0.0534	0.0053

Since it is well known that magnetic dipole transitions in rare earth ions are independent of the ion’s surroundings, the magnetic dipole radiative transition rates *A*^md^ can be evaluated using the following expression:

$$ {A}_{J-J\hbox{'}}^{\mathrm{md}}=\frac{64{\pi}^4{\nu}_{\mathrm{md}}^3}{3h\left(2J+1\right)}{n}^3{S}_{\mathrm{md}} $$

Where *n* is the refractive index, (2*J* + 1) is the degeneracy of the initial state *J* and *v*_md_ is the transition energy of the ^5^D_0_*→*^7^F_1_ transition (cm^−1^*)*, *h* is Planck constant (6.63 × 10^27^ erg s). *S*_md_ is the magnetic dipolar transition line strength, which is independent of host matrix and is equal to 11.26 × 10^−42^ (esu)^2^ cm^2^ [42]. From the definition of the ${A}_{J-{J}^{\prime}}^{\mathrm{md}}$, the refractive index can be calculated to be 1.58.

For a particular transition, the intensity (*I*) of an emission transition is proportional to the radiative decay rate ${A}_{{}{}^7F_J\prime }$, of that transition, which equals the reciprocal of intrinsic lifetime τ_0_. The intensity is also proportional to the area under that emission curve [43]. Thus, the intensity of an emission transition can be written as [44] follows:

$$ I=\eta {\displaystyle \sum_{J^{\hbox{'}}=0,1\dots 6}}{A}_{{}{}^7F_J\hbox{'}} $$

The fluorescence lifetime of the nanoparticles is approximately 1 ms [45,46]. The values of ${A}_{{}{}^7F_J\prime }$, shown in Table 2 were determined by calculating the constant *η*. The radiative branching ratio shown in Table 2 was calculated using ${\beta}_{J-J\hbox{'}}=\frac{A_{J-J\hbox{'}}}{{\displaystyle {\sum}_{J^{\hbox{'}}=0,1\dots 6}}{A}_{J-J\hbox{'}}}$

The electric dipole transitions ^5^D_0_-^7^F_*J′*_ (*J*′ = 2, 4, and 6) can be represented by using the three J-O parameters Ω_*k*_ (*k* = 2, 4, 6) as follows [47,48]:

$$ {A}_{J-J\hbox{'}}^{\mathrm{ed}}=\frac{64{\pi}^4{e}^2\ {\nu}^3n\left({n}^2+2\right)2}{27h\left(2J+1\right)}{\displaystyle \sum_{k=2,4,6}}{\Omega}_k{\left|\psi J\left|{U}^{(k)}\right|\psi \hbox{'}J\hbox{'}\right|}^2 $$

where *h* is the Planck’s constant, *ν* is the transition energy of electric dipole transition (in cm^−1^) and *e* is the charge of an electron, and ${\left|\left\langle {}_{\;}{}^5D_0^{\;}\left|{U}^{(k)}\right|{F}_{J^{\prime }}\right\rangle \right|}^2$ is the double-reduced matrix element. All of the matrix elements for ^5^D_0_ → ^7^F_*J*__′_ transitions are zero [49-51], except those for the ^5^D_0_-^7^F_2_ transition (*U*^(2)^ = 0.0028), the ^5^D_0_-^7^F_4_ transition (*U*^(4)^ = 0.002) and the ^5^D_0_ → ^7^F_6_ transition (*U*^(6)^ = 0.0002). Thus, the values of Ω_*k*_ can be calculated using the emissions of ^5^*D*_0_ → ^7^*F*_*J*'_ (*J*' = 2, 4, 6). The results of our calculations are shown in Table 3 together with the Ω_*k*_ values of Eu^3+^ ions in other hosts [41,51-57].

**Table 3:** **J-O parameters of Eu** ^**3+**^ **in several compounds**

**Compounds**	**Ω**_**2**_ **(10** ^**−20**^ **cm** ^**2**^ **)**	**Ω**_**4**_ **(10** ^**−20**^ **cm** ^**2**^ **)**	**Ω**_**6**_ **(10** ^**−20**^ **cm** ^**2**^ **)**	**Reference**
Gd_2_O_3_:Eu^3+^ nanoplatelets	28.07	1.87	0.05	This work
KLTB:Eu^3+^	14.20	~0	2.40	Saleem (2010) [54]
L4BE:Eu^3+^	17.56	~0	4.26	Babu (2000) [55]
LaOF:Eu^3+^	56.3	13.9	-	Grzyb (2011) [56]
Gd_2_O_3_:Eu^3+^ nanocrystals	5.61	1.57	-	Liu (2006) [57]
Eu_0*.*08_K_0*.*075_Ba_0*.*845_TiO_3_	6.92	1.84	-	Li (2008) [53]
Gd_2_(W_0*.*5_Mo_0*.*5_)O_6_:Eu^3+^	6.91	0.22	-	Yue (2011) [41]
Fluorosilicate glass ceramic	1.38	0.84	-	Zhao (2007) [52]
Fluorophosphate glass	3.24	5.11	2.89	Balda (1996) [51]

These intensity parameters follow the tendency Ω_2_ > Ω_4_ > Ω_6_ found for other materials containing Eu^3+^ ions. It is well known that Ω_2_ is most sensitive to the local structure and its value is indicative the higher asymmetry and higher covalence around the Eu^3+^ ions with their surrounding ligands [58]. However, the parameter Ω_6_ is inversely proportional to the Eu-O band covalency, since it is more strongly affected by the overlap integrals of 4f and 5d orbitals than Ω_2_ and Ω_4_ [58].

### The MRI contrast enhancement

**Figure 8:**
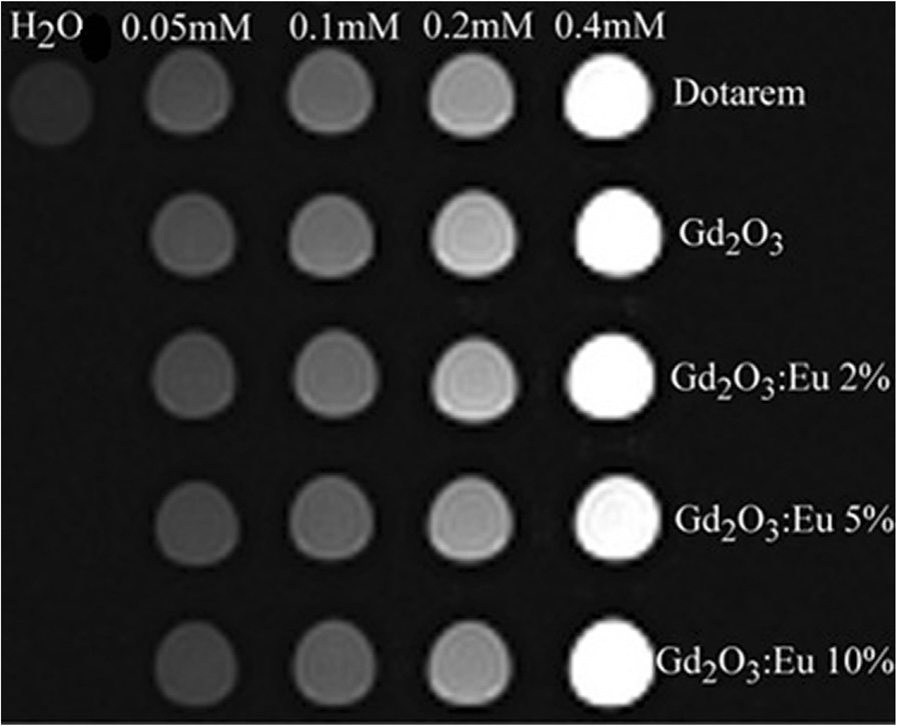
MRI of different concentrations (0.05 to 0.4 mM) of Gd_2_O_3_ and Gd_2_O_3_:Eu 2% to 10% compared to the same concentrations of commercial contrast agent (Dotarem).

**Figure 9:**
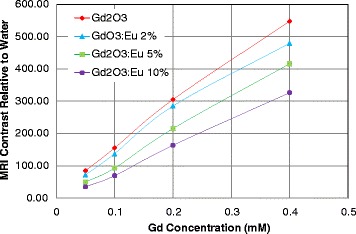
The contrast relative to water due to Gd_2_O_3_ and Gd_2_O_3_: Eu (2% to 10%) for Gd_2_O_3_ molar concentrations from 0.05 to 0.4 mM.

**Figure 10:**
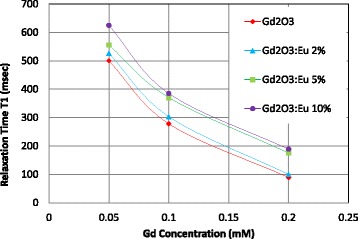
MRI relaxation time (T1) for Gd_2_O_3_ and Gd_2_O_3_:Eu (2% to 10%) for molar concentrations from 0.05 to 0.2 mM.

We have tested MR image enhancement properties of the gadolinium nanoparticles and the Eu-doped nanoparticles using the MRI scanner at King Fahd Specialist Hospital. We also compared the MR images to commercially available MRI contrast agent (DOTAREM) using the same gadolinium concentrations (Gd molar concentrations 0.05, 0.1, 0.2 and 0.4 mM). The gadolinium oxide nanoparticles provided comparable MR image enhancement to the commercially used contrast agent DOTAREM (Figure 8). The addition of Eu reduced the MRI contrast due to the replacement of gadolinium atoms by the Eu atoms in the material structure. Figure 9 shows the contrast relative to water due to Dotarem, Gd_2_O_3_, and Gd_2_O_3_:Eu (2% to 10%) for Gd molar concentration from 0.05 to 0.4 mM. Figure 10 shows the variation of the T1 relaxation time for Dotarem, Gd_2_O_3_, and Gd_2_O_3_:Eu (2% to 10%) for Gd molar concentration from 0.05 to 0.2 mM.

## Conclusions

We synthesized nanoplatelets of Gd_2_O_3_ and Gd_2_O_3_:Eu^3+^ (2%, 5%, and 10%). The doping with Eu preserved the crystalline cubic structure of the Gd_2_O_3_ matrix. The MRI contrast of the Gd_2_O_3_ was comparable to the commercial gadolinium-based contrast agent DOTAREM at the same gadolinium concentrations. Doping the Gd_2_O_3_ with Eu exhibits very strong PL spectra especially in the red region at 612 nm corresponding to the radiative transitions from the Eu-excited state ^5^D_0_ to the ^7^F_2_ state. The strongest red PL was obtained at 5% Eu doping concentration. The stimulated CIE chromaticity coordinates and Judd-Ofelt analysis were used to obtain the radiative properties of the sample from the emission spectra. However, doping with Eu has decreased the MRI contrast and increased the T1 relaxation time. The MRI contrast enhancement decreased with increasing Eu doping concentration due to the replacement of the gadolinium atoms with Eu. The synthesized nanoparticles can be used as a contrast agent for magnetic resonance imaging. The PL in the red region can be exploited in labeling biological materials for fluorescence microscopy applications. The synthesized nanoplatelets have to be coated or encapsulated in biocompatible material such as polyethylene glycol to be used for *in vivo* MRI of cancer tissues with or without targeting molecules.
